# Increased plasma concentration of cell-free DNA precedes disease recurrence in children with high-risk neuroblastoma

**DOI:** 10.1186/s12885-020-6562-8

**Published:** 2020-02-06

**Authors:** Yan Su, Lijun Wang, Chiyi Jiang, Zhixia Yue, Hongjun Fan, Huimin Hong, Chao Duan, Mei Jin, Dawei Zhang, Lihua Qiu, Xianfeng Cheng, Zhong Xu, Xiaoli Ma

**Affiliations:** 1 Beijing Key Laboratory of Pediatric Hematology Oncology, National Discipline of Pediatrics, Ministry of Education, MOE Key Laboratory of Major Diseases in Children, Hematology Oncology Center, Beijing Children’s Hospital, Capital Medical University, National Center for Children’s Health, Beijing, 100045 China; 2 Beijing Keyin Technology Company Limited, Beijing Keyin Evergreen Institutes for Medical Research Company Limited, Eastern Block of Jianwai SOHO, Chaoyang District, Beijing, 100022 China

**Keywords:** Plasma cell free DNA, Neuroblastoma, High risk, Recurrence disease, Molecular marker

## Abstract

**Background:**

Neuroblastoma is the most common extracranial solid tumor of childhood. The high rate of recurrence is associated with a low survival rate for patients with high-risk neuroblastoma. There is thus an urgent need to identify effective predictive biomarkers of disease recurrence.

**Methods:**

A total of 116 patients with high-risk neuroblastoma were recruited at Beijing Children’s Hospital between February 2015 and December 2017. All patients received multidisciplinary treatment, were evaluated for the therapeutic response, and then initiated on maintenance treatment. Blood samples were collected at the beginning of maintenance treatment, every 3 months thereafter, and at the time of disease recurrence. Plasma levels of cell-free DNA (cfDNA) were quantified by qPCR. Receiver operating characteristic (ROC) curve analysis was performed to evaluate the ability of plasma cfDNA concentration to predict recurrence.

**Results:**

Of the 116 patients, 36 (31.0%) developed recurrence during maintenance treatment. The median time to recurrence was 19.00, 9.00, and 8.00 months for patients who had achieved complete response (*n* = 6), partial response (*n* = 25), and stable disease (*n* = 5), respectively, after multidisciplinary treatment. The median plasma cfDNA concentration at the time of recurrence was significantly higher than the concentration in recurrence-free patients throughout maintenance treatment (29.34 ng/mL vs 10.32 ng/mL). Patients recorded a plasma cfDNA level ≥ 29 ng/mL an average of 0.55 months before diagnosis of disease recurrence. ROC analysis of the power of plasma cfDNA to distinguish between patients with or without recurrence yielded an area under the curve of 0.825, with optimal sensitivity and specificity of 80.6 and 71.3%, respectively, at a cfDNA level of 12.93 ng/mL.

**Conclusions:**

High plasma cfDNA concentration is a potential molecular marker to signal disease recurrence in patients with high-risk neuroblastoma.

## Background

Neuroblastoma (NB) originates from neural crest precursor cells of the sympathetic nervous system and is one of the most common pediatric malignancies, accounting for approximately 10% of all childhood cancers [1–3]. About 90% of NB cases occur in children under the age of 5 years [4, 5]. Primary tumors usually arise in the abdomen, but they can also develop in the neck, thorax, and pelvis. Symptoms and signs depend of location of tumor and metastasis [6, 7].

Treatment of NB is based on risk stratification and typically includes surgery, chemotherapy, radiation, and immunotherapy in high risk patients [8–11]. Prevention of tumor recurrence is particularly difficult in patients with high-risk NB, for whom the 5-year survival rate is less than 50% [12–14]. Currently, disease recurrence and metastatic tumor sites are detected by imaging studies and cytological examinations [15]; however, tumor growth is generally advanced at this point. There is thus a great need to identify novel and effective biomarkers to predict NB recurrence.

Plasma cell-free DNA (cfDNA) has become an increasingly attractive potential biomarker for various cancers [16–18]. Typically, cfDNA consists of degraded DNA fragments derived from tumor cells undergoing apoptosis or necrosis. Although such fragments are normally taken up by tissue macrophages, excessive release of DNA from large tumors can result in some reaching the bloodstream. This observation hinted at the possibility that circulating cfDNA could be used to monitor cancer progression [3, 19, 20]. However, little is known about plasma cfDNA concentration in patients with NB or its potential value as a biomarker for disease recurrence.

In the present study, we monitored plasma cfDNA level in patients with high-risk NB during maintenance treatment to determine its relationship to tumor recurrence. We found that a high level of plasma cfDNA preceded disease recurrence and had good discriminatory power, suggesting that it could be used as a molecular marker of NB progression in the clinic.

## Methods

### Patients

A total of 116 patients with high-risk NB were recruited at the Hematology Oncology Center, Beijing Children’s Hospital between February 12,015 and December 312,017. High-risk NB was classified as (i) age older than 18 months and stage IV disease according to the International Neuroblastoma Staging System (INSS); or (ii) any age and stage II–IV disease with N-Myc (*MYCN*) gene amplification. All patients had received multidisciplinary treatment, had been evaluated, and were then started on maintenance treatment. The patients were monitored and evaluated throughout maintenance treatment, with follow-up ending on September 30, 2018. This study and the BCH-NB-2007-HR protocol were approved by the Beijing Children’s Hospital Institutional Ethics Committee (No. 2016–65). Informed consent was obtained from the patients’ parents or guardians. The BCH-NB-2007-HR protocol is based on the Hong Kong Pediatric Hematology and Oncology Study Group guidelines [21] and the results of a study in Germany [22].

### Diagnostic tests and evaluation

Upon initial diagnosis, bone marrow biopsies and/or aspirates were obtained for microscopic examination and identification of NB cells. Genetic abnormalities (amplification of the *MYCN* gene, deletion of the short arm of chromosome 1 [1p36], and/or deletion of the long arm of chromosome 11 [11q23], were detected by fluorescence in situ hybridization. Serum levels of tumor markers, including lactate dehydrogenase (LDH) and neuron-specific enolase (NSE), were quantified.

After multidisciplinary treatment, the therapeutic response was determined by quantification of serum tumor markers, microscopic examination of bone marrow samples, ^131^I- metaiodobenzylguanidine (^131^I-MIBG) scanning, ultrasound, and computed tomography. According to the Response Evaluation Criteria in Solid Tumors (RECIST) criteria, the response was classified as complete remission (CR), partial remission (PR), stable disease (SD), and progressive disease (PD). Patients with CR, PR, or SD entered maintenance treatment.

Quantification of serum tumor markers, microscopic examination of bone marrow, and imaging tests were performed every 3 months, and ^131^I-MIBG scanning was performed every 6 months.

### Treatment

According to the BCH-NB-2007-HR protocol, patients initially diagnosed with high-risk NB received multidisciplinary treatment including induction chemotherapy, surgery, consolidation therapy, and radiotherapy. Some patients received autologous stem-cell transplantation. Common regimens included chemotherapy with high dose cyclophosphamide, adriamycin, and vincristine, chemotherapy with high dose cisplatinum and VP16, surgery after 4–5 cycles of chemotherapy, and harvesting of peripheral blood stem cells for possible autologous hematopoietic stem-cell rescue. The maintenance treatment regimen was 13-cis-retinoic acid 160 mg/m^2^/day on alternate days for 14 days followed by 14 days off treatment for 6–9 months.

### Sample collecting

Blood samples were collected to quantify cfDNA at the beginning of maintenance treatment, every 3 months thereafter, and at the diagnosis of recurrence. Venous blood samples were collected into ethylenediaminetetraacetic acid-coated tubes and centrifuged at 1600×g for 10 min. Supernatants were transferred to fresh tubes and centrifuged at 16,000×g for 10 min. Plasma was removed and stored at − 80 °C until DNA extraction.

### Plasma cfDNA detection

DNA was extracted from 200 μL plasma and eluted in 300 μL elution buffer using QIAmp DNA Blood Mini Kits (Qiagen, Valencia, CA, USA). cfDNA was quantified as previously described [23]. Briefly, DNA was subjected to quantitative polymerase chain reaction (qPCR) using a LightCycler LC 480 PCR (Roche Molecular Systems, Pleasanton, CA, USA). Primers were designed to amplify 79-bp fragments of long interspersed nuclear element 1 (*LINE-1*) DNA, which is derived from apoptotic and non-apoptotic cells. A reference standard curve was established with serial dilutions a standard solution of human genomic DNA (Thermo Fisher Scientific, Waltham, MA, USA). The qPCR reaction mixture contained 2 μL of eluted DNA, 1 μL each of forward and reverse *LINE-1* 79 bp primers (final concentration 0.2 μm), 5 μL of UltraSYBR Mixture (ConWin Biotech, Beijing, China), and 1 μL of double-distilled water. Cycling conditions were 1 min at 95 °C and 35 cycles of 95 °C for 8 s and 60 °C for 15 s. qPCR reactions were performed in triplicate and the mean value was used in calculations. Negative and positive controls (water as template and standard DNA dilutions) were included on each plate. cfDNA concentration was calculated from the standard curve using the 2^-△△Ct^ method.

### Statistics analysis

Data are presented as the median or mean and standard deviation and were analyzed using the Mann–Whitney U test or Chi-square test in R statistical environment (version 3.4.0). Receiver operating characteristic (ROC) curves were constructed and analyzed using the Bioconductor ROC package. A *p* value of < 0.05 was considered significant.

## Results

### Demographic and clinical characteristics

A total of 116 pediatric patients (56 female, 60 male) with high-risk NB were enrolled at the beginning of maintenance treatment (Table 1). Seven patients were younger than 18 months and all of them harbored amplification of the *MYCN* gene. Among the remaining 109 patients, 31 also harbored *MYCN* amplification; 1 had stage III disease and 30 had stage IV disease (INSS classification). At the time of initial diagnosis, 93 (80.2%) of the patients had a primary tumor site in the abdomen, 20 (17.2%) in the thorax, and 3 (2.6%) at other sites. Eighty-eight (75.9%) of the patients had NSE levels < 370 ng/mL; and 14 (12.1%), 88 (75.9%), and 14 (12.1%) had LDH levels of ≤500 IU/L, 500–1500 IU/L, and > 1500 IU/L, respectively. Remarkably, metastasis was detected in one or two organs in 59 patients (50.9%), in three organs in 39 patients (33.6%), and in more than three organs in 18 patients (15.5%). The most frequent metastatic sites were bone, bone marrow, and distant lymph node, which were observed in 72.4, 62.1, and 65.5% of patients, respectively.

**Table 1:** Characteristics of NB patients with high risk

Characteristics	Total cases, *N* (%)
Age (months)	
< 18	7 (6.0)
≥ 18 and < 60	80 (69.0)
≥ 60	29 (25.0)
Sex	
Female	56 (48.3)
Male	60 (51.7)
Primary site	
Abdomen	93 (80.2)
Thorax	20 (17.2)
Other	3 (2.6)
*MYCN* gene	
Amplification	38 (32.8)
Non-amplification	78 (67.2)
NSE (ng/ml)	
< 370	88 (75.9)
≥ 370	28 (24.1)
LDH (IU/L)	
≤ 500	14 (12.1)
> 500 and < 1500	88 (75.9)
≥ 1500	14 (12.1)
Metastatic site	
Bone	84 (72.4)
Bone marrow	72 (62.1)
Distant lymph node	76 (65.5)
Liver	20 (17.2)
Central nervous system	20 (17.2)
Number of organs with metastasis	
< 3	59 (50.9)
3	39 (33.6)
> 3	18 (15.5)

### Recurrence during maintenance treatment

All 116 patients entered maintenance treatment with 13-cis-retinoic acid following evaluation after multidisciplinary treatment. At that time, NB cells were absent from the bone marrow, as confirmed by two independent microscopic examinations; disease progression was confirmed absent by independent radiological experts; serum LDH and NSE levels were decreased; and the ^131^I-MIBG scan was negative. During maintenance treatment, recurrence was diagnosed by a positive microscopic examination of bone marrow and/or in situ or metastatic tumor growth by radiography and ^131^I-MIBG scan. A total of 36 patients (31.0%) developed recurrence during maintenance treatment (Table 2); 5 patients during the first 3 months, 26 patients in the next 6–9 months, and 5 patients at > 12 months, after 13-cis-retinoic acid treatment was stopped.

**Table 2:** Recurrence disease of NB during maintenance treatment

Time point of recurrence	Total cases (*n* = 36)
with 13-cis-retinoic acid	31
the first-three months	5
next 6–9 months	26
after 13-cis-retinoic acid stopped	5
3–6 months	2
6–12 months	2
Over 12 months	1

### Analysis of cfDNA levels in NB patients with and without disease recurrence

**Fig. 1:**
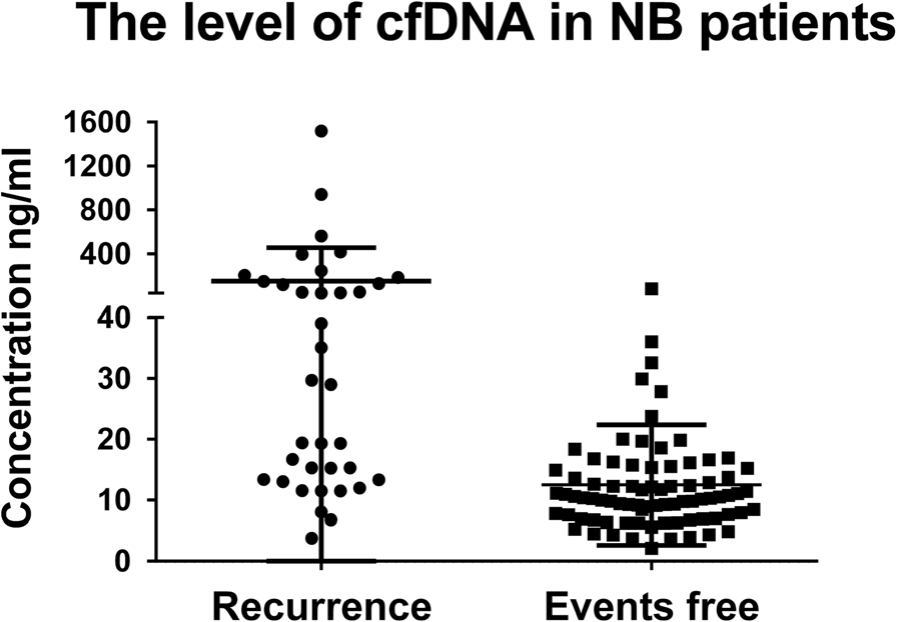
Plasma cfDNA levels in NB patients with or without recurrence during maintenance treatment. Data are presented as the median ± standard deviation of the last measurement before recurrence diagnosis (*n* = 36) or of all measurements throughout maintenance treatment for the recurrence-free group. Each symbol represents an individual patient, *p* < 0.05 by the Mann–Whitney U test

Plasma cfDNA concentrations were measured every 3 months after maintenance treatment initiation until recurrence. Figure 1 shows that the median cfDNA at the time point preceding recurrence (last measurement before diagnosis) was significantly higher than the median cfDNA level throughout maintenance treatment in the recurrence-free group (29.34 ng/mL vs 10.32 ng/mL, *p* < 0.001). In contrast, the cfDNA level in the recurrence group at the start of maintenance treatment was not significantly different from the level in the recurrence-free group throughout maintenance treatment (9.75 ng/mL vs 10.32 ng/mL, *p* > 0.05, Additional file 1: Figure S1).

To evaluate the relationship between the cfDNA level and disease recurrence, we defined a “high” cfDNA level as ≥29.34 ng/mL, which was the median plasma cfDNA level preceding recurrence. The time span between recording a high cfDNA level and disease recurrence ranged from 0 to − 3 months, with a mean value of − 0.55 months (Table 3). Among the 19 patients with high cfDNA levels before recurrence, 10 patients reached the high level within 1 month before recurrence and 9 patients within 1 to 3 months before recurrence. The rise in plasma cfDNA in these patients may be linked to increasing tumor burden, suggesting that plasma cfDNA levels might have utility as a molecular marker to signal disease recurrence.

**Table 3:** Time span between disease recurrce and cfDNA increase in NB patients

Time span between high cfDNA levels and disease recurrence in NB patients	
Patients with cfDNA level > =29.34 ng/ml (n)	19
Time span (Months prededing diagnosis)	−3 < = increase time < =0
Median	0
Average	−0.55
Total patients (n)	19
Time span = 0 (n)	10 (52.6%)
Time span <0 (n)	9 (47.4%)

**Fig. 2:**
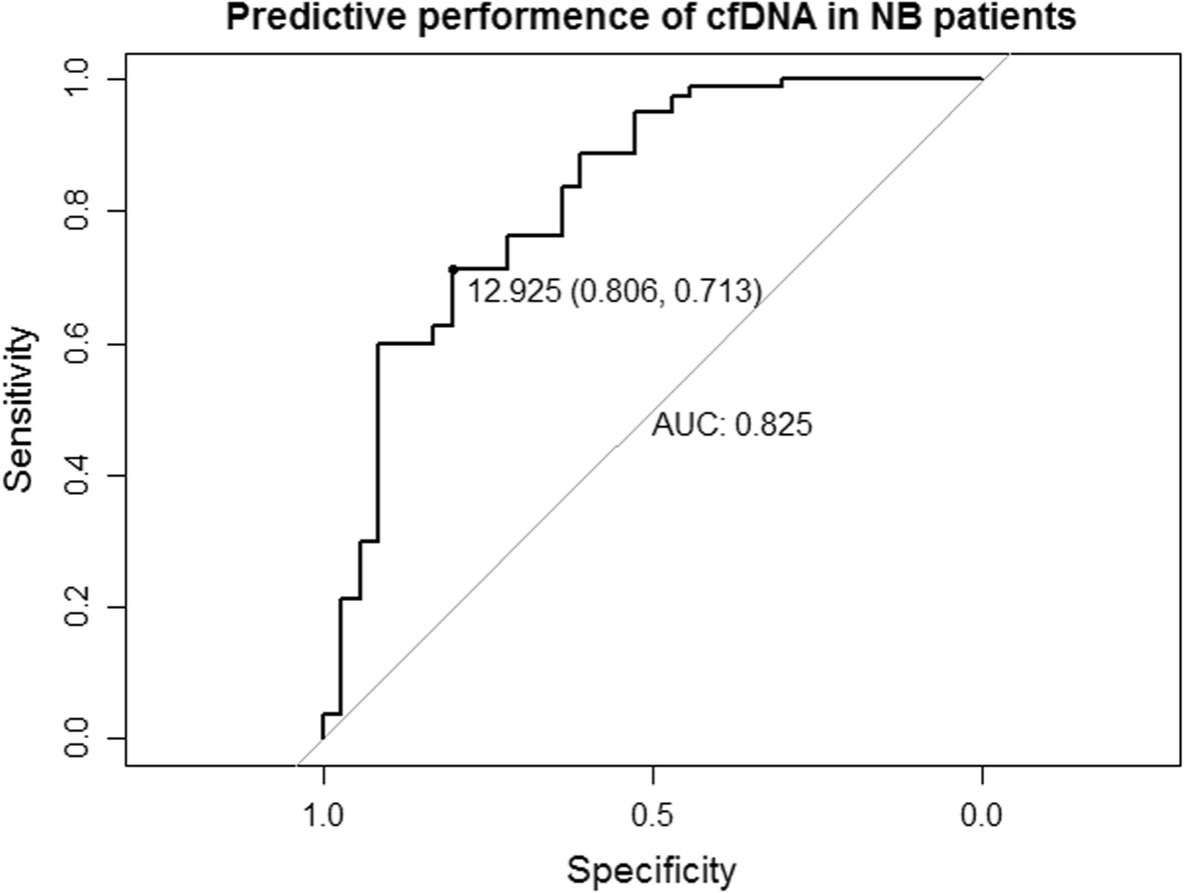
Receiver operating characteristic curve analysis of the predictive value of plasma cfDNA level for NB recurrence. The cfDNA level for optimal sensitivity and specificity and the AUC are indicated

To test this possibility, we performed ROC curve analysis. The area under the ROC curve (AUC) was 0.825, with an optimal sensitivity and specificity of 80.6 and 71.3%, respectively, at a cfDNA concentration of 12.93 ng/mL (Fig. 2). Thus, plasma cfDNA concentration has good discriminatory power for disease recurrence.

### Recurrence among subgroups of NB patients during maintenance treatment

Next, we assessed the rate of recurrence among patient subgroups stratified by their response to initial multidisciplinary treatment (Table 4). Six of 50 (12%) patients in the CR group, 25 of 55 (45.5%) patients in the PR group, and 5 of 11 (45.5%) patients in the SD group experienced disease recurrence during maintenance treatment, indicating a significantly lower recurrence rate for patients who initially achieved CR compared with PR or SD. The time from initiation of maintenance treatment to recurrence was also significantly longer for the CR group than for the PR or SD groups (mean 17.52, 10.97, and 6.64 months, respectively).

**Table 4:** Recurrence among subgroups of NB patients during maintenance treatment

	CR	PR	SD
Total (n)	50	55	11
Mean (months ± SD)	17.52 ± 8.37	10.97 ± 6.81	6.64 ± 5.66
Median (months ± SD)	19.00 ± 8.37	9.00 ± 6.81	8.00 ± 5.66
Recurrence (n)	6 (12%)	25 (45.5%)	5 (45.5%)
Recurrence free (n)	44 (88%)	30 (54.5%)	6 (54.5%)

## Discussion

Neuroblastoma is one of the most common cancers in children, and its incidence has increased by 7% every 10 years between 1985 and 2015 [22, 24]. Advances have been made in the diagnosis and therapy of NB, including better radiological imaging, cytological, biochemical, and molecular techniques; however, the 5-year survival rate of patients with high-risk NB remains below 50% [11, 25, 26]. Many factors contribute to this disappointing outcome, most notably the persistence of chemoresistant minimal residual disease (MRD), which is responsible for disease recurrence in > 50% of patients with high-risk NB [1, 14, 24, 27]. Therefore, accurate detection of MRD is crucial to enable prompt therapeutic action.

Finding biomarkers to evaluate prognosis or response to treatment is an intense area of cancer research. Plasma cfDNA has been extensively investigated as a potential biomarker, especially for malignant metastatic cancers [28]. Compared with tissue-based histological or imaging tests, which are sensitive to sampling bias and poor repeatability, measurement of plasma cfDNA represents a minimally invasive method to monitor tumor burden and thus act as both a clinical and pathological biomarker [29]. cfDNA levels have been shown to be significantly higher in patients with cancer compared with benign disease [30], and is a potential marker of the therapeutic response and prognosis of patients with a wide range of cancers, such as lung cancers and gastrointestinal malignancies [17, 31–33]. cfDNA is thought to originate predominantly from tumor cells and hematopoietic cells [34]. We previously showed that inflammation, transfusion, and therapy with granulocyte-colony stimulating factor are key clinical factors affecting the quantification of cfDNA [35]. To avoid detection of cfDNA from non-tumor cells, blood should not be sampled for tumor cfDNA analysis in these three settings.

Quantification of cfDNA by qPCR has three main advantages over digital PCR and next generation sequencing for detection tumor burden; namely, ease of performance, common use, and low cost. However, its disadvantages include lower sensitivity and detection of a limited number of genomic loci per analysis. Several other methods have been proposed to improve the sensitivity of detection of tumor-derived plasma cfDNA, including gene-specific panel profiling, whole exome/genome sequencing, and digital PCR [36, 37].

Current digital PCR techniques have high sensitivity to detect low allele fractions variants [38]. Whole exome/genome sequencing offers a comprehensive analysis of tumor mutations and has broad applications, but it is an expensive method [39]. Thus, quantification of plasma cfDNA by qPCR represents a relatively simple, inexpensive, and reproducible method to monitor tumor burden.

We previously demonstrated that plasma cfDNA levels correlated strongly with tumor burden in children with NB [23], and could potentially serve as a more effective biomarker than LDH, which is widely used in the clinic. Furthermore, plasma cfDNA concentrations were significantly lower in patients with PR compared with SD, and the concentrations were dynamically associated with changing tumor burden in response to chemotherapy [35]. However, whether cfDNA could serve as an effective molecular marker for recurrence was unknown. Here, we showed that plasma cfDNA levels increased significantly before the diagnosis of recurrence; however, this did not occur in all patients with recurrence, which could be due to a number of factors, including tumor stage, tumor heterogeneity, and other clinicopathological characteristics [19]. The clinical features associated with disease recurrence in NB are complex, and include the metastatic site, tumor cell abundance, and tumor aggressiveness. Therefore, it is not surprising that cfDNA levels vary among individuals with recurrent NB.

Quantification of both cfDNA in plasma and tumor cell-derived mRNA in peripheral blood may be useful for detecting MRD in NB patients [40–42]. In patients with high-risk NB, qPCR-mediated detection of tyrosine hydroxylase (*TH*) and paired-like homeobox 2B (*PHOX2B*) mRNA levels is a sensitive and specific method for detecting MRD [41, 42]. However, whether plasma cfDNA or *TH/PHOX2B* mRNA is the superior marker is difficult to determine. The heterogeneity of NB might suggest that a threshold value of both cfDNA and *TH/PHOX2B* mRNA should be exceeded to declare MRD positivity. Notably, circulating cfDNA may be more stable than mRNA [43], and fewer steps are required for the quantification of cfDNA compared with mRNA, making it less expensive. We are currently investigating the utility of *PHOX2B* mRNA monitoring in NB patients and whether a correlation exists between *PHOX2B* mRNA and cfDNA levels.

## Conclusion

In conclusion, we have shown here that a significant rise in plasma cfDNA concentration occurs between 1 and 3 months before disease recurrence in patients with high-risk NB. Thus, plasma cfDNA could be a promising marker of imminent disease recurrence, or at least a useful monitoring tool, during maintenance treatment for this patient population.

## Supplementary information

**Additional file 1: Figure S1.** Plasma cfDNA levels in NB patients before recurrence. Data are presented as the median ± standard deviation of the measurement at the start of maintenance treatment for the recurrence group (*n* = 36) or of all measurements throughout maintenance treatment for the recurrence-free group (*n* = 80). Each symbol represents an individual patient. Not significant by the Mann–Whitney U test.

## Data Availability

The raw data are available upon reasonable request from the corresponding authors.

## Abbreviations

^131^I-MIBG: ^131^Iodine-metaiodobenzylguanidine
AUC: Area under ROC curve
cfDNA: Cell-free DNA
CR: Complete remission
LDH: Lactate dehydrogenase
MRD: Minimal residual disease
NB: Neuroblastoma
NSE: Neuron-specific enolase
PD: Progressive disease
PR: Partial remission
ROC: Receiver operating characteristic
SD: Stable disease
